# Simple technology for COVID-19 medical solid waste treatment in low-resourced settings

**DOI:** 10.7189/jogh.10.020373

**Published:** 2020-12

**Authors:** Saverio Bellizzi, Sherif A. Kamal, Ahmed E Newir, Giuseppe Pichierri, Paola Salaris, Sergio Pinto, Gabriele Farina, Maura Fiamma, Osama Ali Maher

**Affiliations:** 1 Independent Consultant, Geneva, Switzerland; 2 Building and Construction Department, Faculty of Engineering, October 6 University, Cairo, Egypt; 3 Faculty of Engineering, Mechatronics Department, Faculty of Engineering, , October 6 University, Cairo, Egypt; 4 Kingston Hospital NHS Foundation Trust, Microbiology Department, Kingston upon Thames, UK; 5 Mater Olbia Hospital, Olbia, Italy; 6 University of Cagliari, Cagliari, Italy; 7 University of Sassari, Sassari, Italy; 8 San Francesco Hospital, Nuoro, Italy; 9 Division of Water Resources Engineering, Lund University, Lund, Sweden

A study conducted by The International Council of Nurses (ICN) concluded that there were more than 230 000 infections among health care workers and more than 600 nurses have now died from the coronavirus-19 disease virus [1].

There is not enough analysis on infection rates among health care waste handlers in low- and middle-income countries or countries with no adequate medical solid waste management scheme. In such contexts it is important to explore all possible measures to protect workers under this category from getting infected as a result of exposure to solid waste generated by COVID-19 cases management. The lack of appropriate Personal Protection Equipment (PPEs) prompt us to investigate innovative ways to take advantage of environmental conditions to control virus contamination of medical waste.

While little attention was paid to potential advantages of some environmental conditions in certain countries, it is reported that the COVID-19 can be transmitted largely by garbage collectors [2]. The survival of the SARS-CoV-2 on surfaces has been subject to research, although not conclusive [3].

In this short communication we examine three environmental aspects which affect the survival of similar viruses as a proxy indicator for SARS-CoV-2 survival on surfaces. These atmospheric indicators are represented by Relative Humidity (RH), Air Temperature (AT) and UV Irradiation (UV), which can be taken into consideration to develop some guidance and operations procedures to an intermediate storage unit at health care facilities for COVID-19, in preparation for a final safe disposal among municipal waste.

Duan et al. in their study around deactivation of SARS CoV-P9 in 2003 showed total destruction of virus infectivity when exposed to UV (260 nM) for about 60 minutes [4]. The same study pointed out that the virus was totally deactivated after 90, 60, 30 minutes at 56°C, 67°C and 75°C respectively. Another study on SARS-CoV-2 concluded that the virus can survive on different surfaces from hours to days and the optimum deactivation of the virus happened at around 50% RH [5]. The same study confirmed that the virus infectivity reduced with increased temperatures (higher than 40°C).

The mechanisms whereby RH, AT and UV inactivate coronaviruses are mostly through damage of the outer membrane and the Spike (S) protein. Specifically, the three-dimensional structure of membranes is disrupted by atmosphere heat, moisture and UV.

UV irradiation is a highly effective method to inactivate the new corona virus SARS-CoV-2, even at the higher viral load levels that are found in research laboratories. Different independent studies have demonstrated how SARS-CoV-2 could effectively be inactivated by UVC irradiation whereas UVA-irradiation was much less effective [6,7]. On the other hand, a very recent investigation has showed that SARS-CoV-2 decayed more rapidly when either humidity or temperature was increased but that droplet volume (1 to 50 μl) and surface type (stainless steel, plastic, or nitrile glove) did not significantly impact decay rate. Important to note how the virus half-life range between 6.3 and 18.6 hours at 24°C was reduced to 1.0 to 8.9 h when the temperature was increased to 35°C [8].

**Figure:**
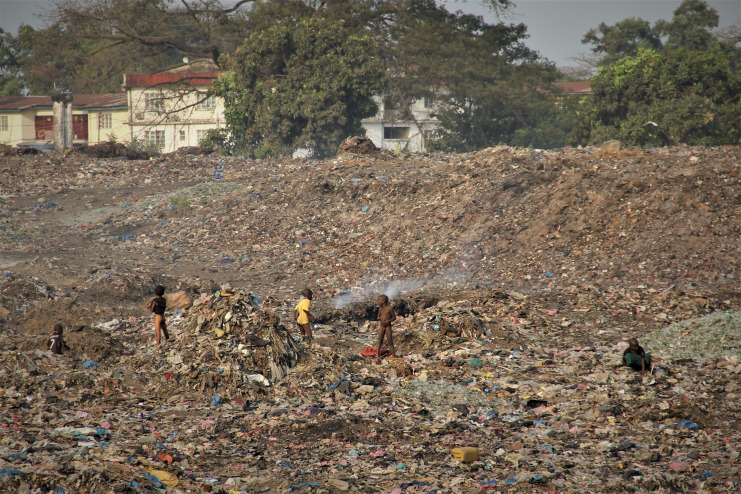
Photo: From Saverio Bellizzi’s own collection (used with permission).

In countries with abundance of solar radiation and long sunny hours, it is easy to achieve these favorable conditions for virus inactivation with simple technology. The proposed technology here depends on producing a simple collection system based on minimum protection procedures and low costing.

A temperature above 40°C can be obtained during the summer in many developing countries such as the WHO Easter Mediterranean Region where sunny hours per year can exceed 40°C. Achieving this temperature inside a transparent plastic box can be feasible and will allow medical waste to be exposed for an adequate length of time to UV. Natural Ventilation can be a good tool to generate the favorable conditions for RH of 50% [9].

The majority of research in the area of occupational health and the management of research so far have been concentrated on countries with systems where the management of hazardous waste and its regulations is well established. For many developing countries, medical waste is frequently mixed with municipal waste in the final disposal. We are calling on developing countries to utilize available environmental conditions to have a simple approach for making an interim treatment of medical waste generated from the management of COVID-19 patients. This approach would be very simple to deal with and would contribute to better protection of the health care workers in these countries.

While effective vaccines and therapeutics are under development, mitigating the transmission of SARS-CoV-2 also through safe waste disposal is critically important to reduce the number of COVID-19 cases. In this context, understanding the role of low-cost measures to deactivate the virus via UV, AT and RH will enable potentially high-impact mitigation strategies.
